# Fibrinogen-to-albumin ratio as a novel biomarker in pneumoconiosis: a retrospective cohort study

**DOI:** 10.13075/ijomeh.1896.02711

**Published:** 2026

**Authors:** Seval Müzeyyen Ecin, Semra Özkan

**Affiliations:** 1 Mersin City Training and Research Hospital, Unit of Occupational Diseases Clinic, Mersin, Turkey; 2 Mersin City Training and Research Hospital, Unit of Internal Medicine Clinic, Mersin, Turkey

**Keywords:** pneumoconiosis, fibrinogen-to-albumin ratio, occupational dust exposure, systemic inflammation, biomarkers, workers

## Abstract

**Objectives:**

Pneumoconiosis is the most common occupational respiratory disease in Turkey and is associated with chronic systemic inflammation. Both occupational dust exposure and heavy metals such as lead may contribute to inflammatory responses. Novel inflammatory indices, including the fibrinogen-to-albumin ratio (FAR), have gained attention; however, their supportive role in occupational lung diseases remains insufficiently defined.

**Material and Methods:**

This retrospective study included 156 workers who presented to an Occupational Diseases Outpatient Clinic in Mersin, Turkey, in 2019–2023. Demographic, occupational, hematological, biochemical, and inflammatory parameters were collected. Pneumoconiosis was diagnosed based on occupational silica exposure and high-resolution computed tomography findings. Univariable and multivariable logistic regression analyses were performed to evaluate associations between pneumoconiosis and selected biomarkers. Receiver operating characteristic analysis was used to assess the discriminative performance of FAR.

**Results:**

Thirty-two workers (20.5%) were diagnosed with early-stage pneumoconiosis. Glass workers represented the largest subgroup (37.5%). Compared with non-pneumoconiosis participants, pneumoconiosis patients were older and had longer work duration, higher hemoglobin, fibrinogen, blood lead levels, and FAR, as well as lower platelet count, albumin, and glomerular filtration rate. In univariable analyses, lower albumin and higher fibrinogen and FAR were associated with pneumoconiosis. In multivariable analysis, only FAR remained independently associated with pneumoconiosis (OR = 1.1, 95% CI: 1.0–1.3, p = 0.004). Receiver operating characteristic analysis demonstrated that a FAR cut-off value >69.9 predicted pneumoconiosis with 73.9% sensitivity and 72.1% specificity (AUC = 0.741).

**Conclusions:**

Fibrinogen-to-albumin ratio was independently associated with pneumoconiosis, whereas albumin, fibrinogen, and blood lead did not retain significance after adjustment. Given its moderate discriminative ability, FAR should be considered a supportive and complementary biomarker reflecting systemic inflammation rather than a stand-alone diagnostic tool. Larger multicenter prospective studies are warranted to validate these findings.

## Highlights

- Fibrinogen-to-albumin ratio (FAR) was independently associated with pneumoconiosis.
- Patients with pneumoconiosis had approx. 17% higher FAR values.
- Fibrinogen-to-albumin ratio showed moderate discrimination and may support occupational health surveillance.

## INTRODUCTION

Occupational diseases are health conditions resulting from exposure to risk factors in the workplace [1]. Globally, occupational diseases include a wide spectrum of conditions affecting different organ systems [2], while in Turkey, respiratory diseases predominate, with pneumoconiosis being the most frequent subtype [3]. Pneumoconiosis remains a major public health problem in countries with ongoing occupational dust exposure, particularly in mining, ceramics, cement, and glass industries.

Occupational exposure to inorganic dusts, chemicals, and heavy metals activates systemic and pulmonary inflammatory pathways and may lead to chronic respiratory diseases such as pneumoconiosis and asthma [4]. Pneumoconioses are parenchymal lung diseases caused by inhalation of inorganic dusts and are often asymptomatic in early stages, but may progress to chronic respiratory impairment over time [5]. Diagnosis relies on occupational exposure history combined with radiological evaluation, with high-resolution computed tomography (HRCT) widely used due to its high sensitivity for early parenchymal changes [5, 6]. Occupations at highest risk include mining, quarrying, tunnel construction, foundries, ceramics, and cement production [7].

Lead is one of the most commonly detected heavy metals in occupational settings, with exposure occurring in industries such as smelting, battery recycling, ceramics, and printing [8]. Workers in glass, crystal, and ceramic production may also show elevated blood lead levels due to dust exposure [9]. Long-term lead exposure is associated with hypertension, anemia, neurotoxicity, nephrotoxicity, and impaired liver function [8]. Importantly, lead exposure may also contribute to systemic inflammation through oxidative stress, altered hepatic protein synthesis, increased acute-phase reactants such as fibrinogen, and reduced albumin levels via inflammatory and metabolic pathways [8, 9].

Both pneumoconiosis and lead exposure are therefore linked to systemic inflammation. In this context, composite inflammatory indices such as the fibrinogen-to-albumin ratio (FAR), neutrophil-to-albumin ratio (NAR), and C-reactive protein (CRP)-to-albumin ratio (CAR) have recently gained attention as markers of systemic inflammatory burden in various clinical conditions [10–12]. Fibrinogen-to-albumin ratio, in particular, has been studied as an indicator of malnutrition, coagulation abnormalities, chronic inflammation, liver dysfunction, and malignancy [13–16]. However, the relevance of these composite inflammatory indices in occupational lung diseases, including pneumoconiosis, remains insufficiently explored.

Neutrophil-to-albumin ratio and CAR were included as comparative inflammatory indices because they are established markers of systemic inflammation and allow assessment of whether FAR provides additional or complementary information in the context of occupational disease [10–12]. Despite extensive research on the respiratory effects of dust exposure and the systemic consequences of lead toxicity, no previous studies have specifically evaluated FAR, NAR, or CAR in patients with pneumoconiosis, highlighting a clear gap in the existing literature.

It should be emphasized that FAR is not a disease-specific biomarker, but rather reflects systemic inflammation potentially influenced by occupational dust exposure and related pathophysiological processes. Clarifying these relationships may provide supportive tools for risk stratification and monitoring in occupational health practice.

The present study included patients who reported to the Occupational Diseases Outpatient Clinic in Mersin, Turkey, in 2019–2023 with suspected occupational disease. The authors compared patients diagnosed with pneumoconiosis and workers without pneumoconiosis in terms of demographic characteristics, occupational background, hematological and biochemical parameters, FAR, NAR, CAR, and blood lead levels to investigate their potential association with pneumoconiosis.

## MATERIAL AND METHODS

### Study design and participants

Patients who reported to the Occupational Diseases Outpatient Clinic in Mersin, Turkey, with suspected occupational diseases between April 1, 2019 – June 30, 2023, were included. This study was designed as a retrospective observational cohort study. Eligible participants were adults aged 18–90 years with accessible records and without a history of malnutrition, coagulation disorders, systemic inflammation, or liver failure. Patients outside this age range, those with the aforementioned conditions, or those with incomplete records were excluded. Data were retrospectively collected from the hospital information system between September 15 – November 15, 2023.

### Clinical and radiological assessment

All patients underwent evaluation by an occupational diseases subspecialist, including hematological and biochemical tests, chest X-ray, HRCT, and pulmonary function tests. Of the 156 patients, 60 (38.5%) had no detectable disease and were classified as healthy workers. Pneumoconiosis was diagnosed based on a history of occupational silica exposure and the presence of characteristic bilateral, well-defined nodules in centrilobular and subpleural distributions on HRCT [5, 6]. Radiological assessments were performed independently and blinded to laboratory results. A total of 32 patients (20.5%) met these criteria and were classified as having pneumoconiosis.

Other occupational diseases were identified in 34 patients (21.8%), including asthma, hearing loss, dermatitis, discopathy, heavy metal elevation, and latex allergy, using standardized diagnostic criteria [17–21].

### Laboratory and biochemical analyses

Demographic data (age, sex, work duration), clinical diagnoses, and laboratory results were collected. Laboratory analyses included complete blood count, fasting blood glucose, urea, creatinine, lipid profile, liver enzymes, bilirubin, lactate dehydrogenase (LDH), γ-glutamyl transferase (GGT), uric acid, electrolytes, and blood lead levels. All laboratory analyses were performed in the central hospital laboratory using standardized and validated automated analyzers. Complete blood count parameters were measured using an automated hematology analyzer. Biochemical parameters, including liver and kidney function tests, lipid profile, and electrolytes, were analyzed using automated clinical chemistry analyzers. Serum fibrinogen levels were measured using a clot-based coagulation method, CRP levels were determined by immunoturbidimetric assays, and blood lead concentrations were measured by atomic absorption spectrometry in accordance with standard laboratory protocols.

Inflammatory indices were calculated as follows [10–12]:


(1) FAR=fibrinogen/albumin

(2) NAR=neutrophil count/albumin

(3) CAR=CRP/albumin

Comparisons were performed between healthy workers and pneumoconiosis patients.

### Statistical analysis

All analyses were performed using SPSS v. 21.0 (IBM Corp., Armonk, NY, USA). Normality of continuous variables was tested using the Kolmogorov–Smirnov test. Data were expressed as mean (M) ± standard deviation (SD) for normally distributed variables and median (range) for non-normally distributed variables. Categorical variables were presented as frequencies and percentages. Comparisons between groups were performed using the independent samples t-test or Mann-Whitney U test for continuous variables and the χ^2^ or Fisher's exact test for categorical variables.

Univariable and multivariable logistic regression analyses (adjusted for age and sex) were used to evaluate associations between pneumoconiosis and selected variables (albumin, blood lead, and FAR). Patients with pneumoconiosis were compared with all participants without pneumoconiosis, including both healthy workers and those diagnosed with other occupational diseases. This reference group was chosen to reflect real-world occupational health practice, where pneumoconiosis must be differentiated from a heterogeneous population presenting with suspected occupational disease.

Potential confounders such as smoking status, work duration, BMI, and comorbidities were considered; however, these variables were not included in the multivariable model due to incomplete data availability and the limited number of pneumoconiosis cases, in order to avoid model overfitting. Due to mathematical coupling, fibrinogen and albumin were not simultaneously included in multivariable models with FAR.

Receiver operating characteristic (ROC) curve analysis was conducted to determine the optimal FAR cut-off value for pneumoconiosis. The ROC analysis was used to assess the discriminative performance of FAR as a supportive biomarker rather than as a replacement for radiological diagnosis, which remains the gold standard. Sensitivity, specificity, and likelihood ratios were calculated. A 2-tailed p-value ≤0.05 was considered statistically significant.

### Ethical considerations

The study was conducted in accordance with the principles of the Declaration of Helsinki. Ethical approval was obtained from the Non-Interventional Clinical Ethics Committee of Mersin University (decision dated September 6, 2023; approval No. 2023/554). Due to the retrospective design, informed consent was waived.

## RESULTS

A total of 156 patients presented to the Occupational Diseases Outpatient Clinic with a prediagnosis of occupational disease in 2019–2023. The age of the study population was M±SD 42.0±8.5 years, and 144 patients (92.3%) were male. The largest occupational group was glass workers (N = 43, 27.6%), followed by cement workers (N = 26, 16.7%) and welders (N = 16, 10.3%). Among all patients, 77 (49.4%) were referred with suspected pneumoconiosis and 31 (19.9%) with suspected asthma. Notably, 60 patients (38.5%) were found to have no occupational disease after comprehensive evaluation.

Pneumoconiosis was the most frequently confirmed diagnosis, identified in 32 patients (20.5%), all of whom were diagnosed with silicosis, while no cases of asbestosis were detected. Among the 77 workers referred with a prediagnosis of pneumoconiosis, 35 (45.5%) were employed in the glass industry, 13 (16.9%) in the cement industry, and 5 (6.5%) in mining. Of the 32 patients in whom pneumoconiosis was confirmed, 12 (37.5%) were glass workers, 4 (12.5%) were cement workers, and 2 (6.3%) were miners, indicating a predominance of silica-exposed occupations.

Asthma was diagnosed in 25 patients (16.0%), while other lung pathologies were identified in 7 (4.5%) patients. All pneumoconiosis cases were classified as early-stage disease based on radiological findings. The prevalence of smoking was 50% among patients with pneumoconiosis and 39.1% among those without pneumoconiosis. The most common reason for presentation was referral by a workplace physician, documented in 90 (57.7%) patients. Demographic, occupational, and baseline laboratory characteristics of the study population are summarized in Table 1.

**Table 1.** Demographic, laboratory data and occupational disease characteristics of patients who reported to the Occupational Diseases Outpatient Clinic with suspected occupational diseases, April 1, 2019 – June 30, 2023, Turkey

Variable	Participants (N = 156)
Socioeconomic	
age [years] (M±SD)	42.0±8.5
sex [n (%)]	
male	144 (92.3)
female	12 (7.7)
current smoking [n (%)]	61 (39.1)
working time [years] (M±SD)	16.1±9.1
occupation [n (%)]	
glass worker	43 (27.6)
cement worker	26 (16.7)
cleaning worker	10 (6.4)
miner	6 (3.8)
welder	16 (10.3)
nurse	7 (4.5)
furniture worker	16 (10.3)
food worker	5 (3.2)
machine worker	12 (7.7)
textile worker	6 (3.8)
blue collar	6 (3.8)
officer	12 (7.7)
Medical	
pre-diagnosis [n (%)]	
pneumoconiosis	77 (49.4)
asthma	31 (19.9)
other lung pathologies	6 (3.8)
dermatitis	7 (4.5)
latex allergy	6 (3.8)
hearing loss	13 (8.3)
diabetes mellitus	1 (0.6)
discopathy	8 (5.1)
heavy metal exposure	5 (3.2)
lung cancer	2 (1.3)
diagnosis [n (%)]	
no disease^a^	60 (38.5)
pneumoconiosis^b^	32 (20.5)
asthma	25 (16.0)
other lung pathologies	7 (4.5)
dermatitis	7 (4.5)
latex allergy	6 (3.8)
hearing loss	7 (4.5)
diabetes mellitus	1 (0.6)
discopathy	7 (4.5)
heavy metal exposure	3 (1.9)
lung cancer	1 (0.6)
Other	
application [n (%)]	
personal	48 (30.8)
occupational physician	90 (57.7)
other doctors	18 (11.5)

a Patients with no disease detected.

b Patients with a history of occupational exposure to pneumoconiotic substances and pneumoconiotic nodules on CT.

Patients diagnosed with any occupational disease were significantly more likely to present with clinical complaints compared with those without occupational disease (79.2% vs. 20.0%, p < 0.001). In addition, patients with occupational diseases had shorter work duration (p = 0.050), lower alanine aminotransferase levels (p = 0.020), sodium levels (p = 0.020), and albumin levels (p = 0.050), higher blood lead levels (p = 0.050) and CRP levels (p = 0.040) compared with patients in whom no occupational disease was detected.

When patients with pneumoconiosis were compared with those without pneumoconiosis, several significant differences were observed. Patients with pneumoconiosis were older (M±SD 46.1±6.9 years, vs. 40.9±8.5 years, p < 0.001) and had a longer work duration (M±SD 20.8±7.7 years, vs. 14.9±9.0 years, p = 0.010). Hematological and biochemical analyses revealed higher hemoglobin levels (p = 0.020), higher mean corpuscular volume (p = 0.050), higher fibrinogen levels (p = 0.020), higher blood lead levels (p = 0.050), and lower platelet counts (p = 0.020) in pneumoconiosis patients. In addition, glomerular filtration rate (GFR) and serum albumin levels were significantly lower in patients with pneumoconiosis (p = 0.010 and p = 0.010, respectively).

Regarding inflammatory indices, patients with pneumoconiosis had significantly higher FAR values compared with non-pneumoconiosis participants (M±SD 74.7±12.7 vs. 63.7±15.9, p = 0.030). This corresponded to an approx. 17% higher mean FAR value in the pneumoconiosis group. Fibrinogen-to-albumin ratio values were M±SD 74.7±12.7 in pneumoconiosis patients, 60.5±17.1 in patients with other occupational diseases, and 65.8±14.7 in healthy workers. Detailed comparisons between pneumoconiosis and non-pneumoconiosis groups are presented in Table 2.

**Table 2.** Relationship between demographic and laboratory data in occupational diseases and pneumoconiosis in patients who reported to the Occupational Diseases Outpatient Clinic with suspected occupational diseases, April 1, 2019 – June 30, 2023, Turkey

Variable	Participants (N = 156)		p	Participants (N = 156)		p
	with occupational disease (N = 96)	without disease^a^ (N = 60)		with pneumoconiosis^b^ (N = 32)	without pneumoconiosis (N = 124)	
Socioeconomic						
age [years] (M±SD)	40.9±8.6	43.6±7.9	**0.060**	46.1±6.9	40.9±8.5	**<0.001**
sex (male) [n (%)]	86 (89.6)	58 (96.7)	**0.100**	32 (100)	112 (90.3)	**0.070**
complaint [n (%)]	76 (79.2)	12 (20.0)	**<0.001**	19 (59.4)	69 (55.6)	**0.800**
working time [years] (M±SD)	15.0±9.0	17.9±8.9	**0.050**	20.8±7.7	14.9±9.0	**0.010**
Laboratory results						
WBC [×10³/µl] (M±SD)	7.9±1.9	7.7±1.8	**0.600**	7.6±1.8	7.9±1.9	**0.400**
neutrophil [×10³/µl] (M±SD)	4.9±1.7	4.7±1.4	**0.700**	4.5±1.7	4.9±1.6	**0.400**
lymphocyte [×10³/µl] (M±SD)	2.1±0.6	2.2±0.5	**0.900**	2.1±0.6	2.2±0.6	**0.800**
hemoglobin [g/dl] (M±SD)	14.8±1.4	15.1±1.2	**0.100**	15.4±0.9	14.9±1.4	**0.020**
hematocrit [%] (M±SD)	48.2±4.9	44.6±3.0	0.500	45.9±3.5	43.5±3.5	0.300
MCV [fl] (M±SD)	86.8±6.3	86.6±6.1	**0.800**	88.7±3.7	86.2±6.6	**0.050**
platelet [×10³/µl] (M±SD)	243.7±60.5	253.6±51.8	**0.300**	230.5±37.3	252.2±60.5	**0.020**
MPV [fl] (M±SD)	8.8±0.9	8.9±0.8	**0.500**	9.0±0.8	8.8±0.9	**0.400**
glucose [mg/dl] (M±SD)	100.7±19.6	99.9±14.6	**0.800**	97.5±10.3	101.4±19.2	**0.200**
creatinine [mg/dl] (M±SD)	0.8±0.2	0.9±0.1	**0.200**	0.8±0.2	0.8±0.1	**0.100**
GFR [ml/min/1.73 m^2^] (M±SD)	107.7±14.3	104.7±12.2	**0.200**	100.6±13.6	108.0±13.1	**0.010**
ALT [U/l] (M±SD)	24.6±11.3	29.9±14.4	**0.020**	23.6±9.2	27.6±13.6	**0.100**
albumin [g/dl] (M±SD)	4.5±0.2	4.6±0.2	**0.050**	4.5±0.2	4.6±0.2	**0.010**
lead [µg/dl] (M±SD)	2.9±3.0	1.8±1.4	**0.050**	3.2±3.2	1.8±1.4	**0.050**
CRP [mg/dl] (M±SD)	0.5±0.4	0.4±0.1	**0.040**	0.5±0.3	0.4±0.3	**0.400**
ferritin (Me (min.–max))	57.4 (4.2–366)	50.8 (4.3–294)	**0.900**	64.0 (24.7–346)	51.4 (4.2–366)	**0.900**
fibrinogen [mg/dl] (M±SD)	303.5±70.4	306.3±67.2	**0.800**	336.8±57.8	294.4±69.0	**0.020**
NAR (M±SD)	1.02±0.40	1.01±0.30	0.900	1.04±0.41	1.01±0.30	0.900
CAR (M±SD)	0.10±0.08	0.07±0.03	**0.060**	0.09±0.07	0.09±0.07	**0.500**
FAR (M±SD)	66.9±16.7	65.8±14.7	**0.700**	74.7±12.7	63.7±15.9	**0.030**

ALT – alanine aminotransferase; AST – aspartate transaminase; CAR – CRP-to-albumin ratio; CRP – C-reactive protein; GFR – glomerular filtration rate; FAR – fibrinogen-to-albumin ratio; HB – hemoglobin; LDH – lactate dehydrogenase; MCV – mean corpuscular volume; MPV – mean platelet volume; NAR – neutrophil-to-albumin ratio; WBC – white blood cell.

Bolded are statistically significant values.

All p-values are presented with 3 decimal places; p-values <0.001 are reported as p < 0.001.

a Patients with no disease detected.

b Patients with a history of occupational exposure to pneumoconiotic substances and pneumoconiotic nodules on CT.

In age- and sex-adjusted logistic regression analyses, lower albumin levels (OR = 0.04, 95% CI: 0.03–0.50, p = 0.010), higher fibrinogen levels (OR = 1.0, 95% CI: 1.0–1.1, p = 0.010), and higher FAR values (OR = 1.1, 95% CI: 1.0–1.1, p = 0.006) were significantly associated with pneumoconiosis in univariable models. In multivariable analysis, only FAR remained independently associated with pneumoconiosis (OR = 1.1, 95% CI: 1.0–1.3, p = 0.004), whereas albumin, fibrinogen, and blood lead levels did not retain statistical significance (Table 3).

**Table 3.** The age-and sex-adjusted univariable and multivariable logistic regression analysis between pneumoconiosis and albumin, lead, and fibrinogen-to-albumin ratio (FAR) in patients who reported to the Occupational Diseases Outpatient Clinic with suspected occupational diseases, April 1, 2019 – June 30, 2023, Turkey

Variable	Univariable OR (95% CI)	p	Multivariable OR (95% CI)	p
Age	1.1 (1.0–1.1)	**0.007**	1.1 (0.9–1.2)	**0.300**
Sex^a^	–	–	–	–
Albumin	0.04 (0.03–0.50)	**0.010**	0.02 (0.0–1.5)	**0.800**
Lead	1.1 (0.9–1.1)	**0.200**	1.2 (0.9–1.4)	**0.200**
Fibrinogen	1.0 (1.0–1.1)	**0.010**	–	–
FAR	1.1 (1.0–1.1)	**0.006**	1.1 (1.0–1.3)	**0.004**

Fibrinogen and albumin were not simultaneously included in the multivariable model with FAR due to mathematical coupling and potential collinearity.

The reference group for logistic regression analyses consisted of all participants without pneumoconiosis, including healthy workers and those diagnosed with other occupational diseases.

Bolded values indicate statistically significant associations (p < 0.05).

All p-values are presented with 3 decimal places; p-values < 0.001 are reported as p < 0.001.

The dashes indicate variables that were not included in the multivariable model due to collinearity or because they were used as reference variables.

a Sex was included as an adjustment variable in the multivariable model; odds ratios are not shown due to sparse data.

**Figure 1.**
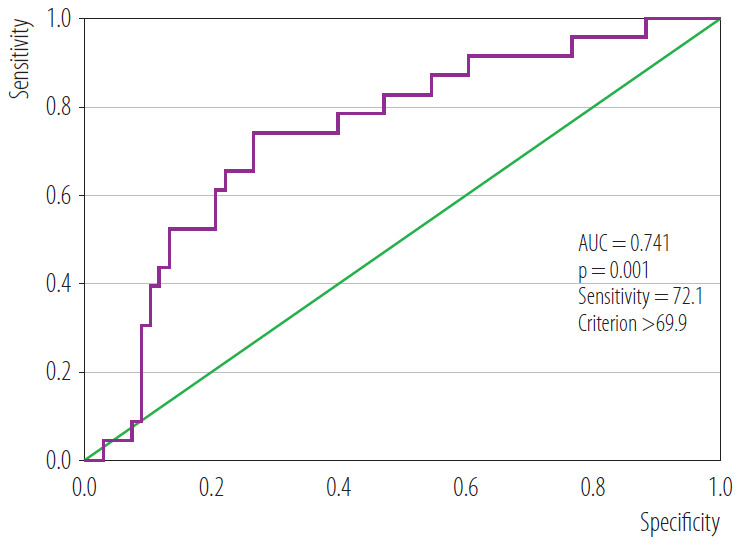
Receiver operating characteristic (ROC) curve of fibrinogen-to-albumin ratio (FAR) for predicting pneumoconiosis in patients who reported to the Occupational Diseases Outpatient Clinic with suspected occupational diseases, April 1, 2019 – June 30, 2023, Turkey

Receiver operating characteristic curve analysis demonstrated that a FAR cut-off value >69.9 predicted pneumoconiosis with a sensitivity of 73.9% and a specificity of 72.1%. The area under the curve (AUC) was 0.741 (95% CI: 0.635–0.857, p = 0.001). The positive likelihood ratio was 2.6, and the negative likelihood ratio was 0.36. These findings indicate a moderate discriminative performance of FAR for distinguishing pneumoconiosis from non-pneumoconiosis cases. The ROC curve for FAR is shown in Figure 1.

AUC – area under the curve.

## DISCUSSION

This study demonstrated that the FAR was independently associated with pneumoconiosis in workers evaluated for suspected occupational diseases. Patients with pneumoconiosis exhibited significantly higher FAR values than non-pneumoconiosis participants, and FAR remained an independent predictor after adjustment for age and sex. These findings suggest that FAR reflects systemic inflammatory processes related to long-term occupational dust exposure and early-stage pneumoconiosis.

Pneumoconiosis was the most frequently diagnosed occupational disease in the authors' clinic, consistent with national data indicating that respiratory diseases remain the leading occupational conditions in Turkey [21]. To maintain focus on the main findings, detailed discussion of referral pathways, occupational health regulations, and global epidemiological comparisons has been condensed. In the authors' cohort, glass workers constituted the largest affected group, which is consistent with the high silica exposure risk in this sector [22]. All pneumoconiosis patients were male, with a mean employment duration >20 years, in line with the long latency period required for radiological manifestation of the disease [23, 24]. Smoking prevalence was high and may have contributed to hematological alterations; however, chronic oxidative stress and persistent inflammation induced by dust exposure remain the central mechanisms in pneumoconiosis pathogenesis [25–27].

Albumin is a negative acute-phase reactant and a marker of nutritional and inflammatory status. Previous studies have reported reduced albumin levels in pneumoconiosis patients [28, 29], and similar findings were observed in the authors' cohort. However, albumin did not remain an independent predictor after adjustment, suggesting that its association may be confounded by broader inflammatory and occupational factors.

Fibrinogen, a positive acute-phase protein, was significantly elevated in pneumoconiosis patients in univariable analyses, consistent with earlier reports in coal workers [30, 31]. Nevertheless, fibrinogen lost significance in multivariable models, indicating that its inflammatory signal may be better captured when interpreted in combination with albumin rather than as an isolated marker.

The FAR, integrating 2 opposing acute-phase proteins, has emerged as a composite marker of systemic inflammation, coagulation imbalance, and adverse prognosis in various chronic inflammatory conditions, including cardiovascular diseases, malignancies, and systemic infections [10,14–17]. In the present study, FAR showed the strongest and most consistent association with pneumoconiosis. Beyond statistical significance, the approx. 17% higher FAR observed in pneumoconiosis patients compared with non-pneumoconiosis participants suggests a potentially clinically meaningful difference. Previous literature indicates that even modest elevations in FAR may reflect chronic low-grade inflammatory burden and disease severity [13–16]. In the context of pneumoconiosis, where prolonged dust exposure induces sustained inflammatory and oxidative pathways [27,30,31], FAR may capture subclinical systemic inflammation not fully reflected by single laboratory parameters.

Receiver operating characteristic analysis demonstrated a FAR cut-off value >69.9 with moderate diagnostic accuracy. However, the observed odds ratio (1.1) and AUC value (0.741) indicate only moderate discriminative ability. Therefore, FAR should not be interpreted as a definitive diagnostic marker for pneumoconiosis.

Importantly, FAR cannot and should not be considered a substitute for HRCT, which remains the gold standard for pneumoconiosis diagnosis [5–7]. Rather, FAR may serve as a complementary biomarker reflecting systemic inflammatory burden associated with occupational dust exposure. From a practical perspective, FAR may support risk stratification, screening of high-risk workers, and longitudinal monitoring within occupational health surveillance programs, particularly when interpreted alongside clinical evaluation and radiological findings.

This study has several strengths. To the best of the authors' knowledge, it is the first study to evaluate FAR, together with NAR and CAR, in patients with pneumoconiosis, addressing a clear gap in the literature. Diagnostic accuracy was strengthened by standardized HRCT evaluation, and the study reflects real-world occupational health practice.

Several limitations should be acknowledged. The retrospective, single-center design limits generalizability and may introduce selection bias. The relatively small sample size reduced statistical power and precluded sensitivity analyses restricted to healthy controls only. The reference group included both healthy workers and individuals with other occupational diseases, which may have diluted the disease-specific discriminatory capacity of FAR. In addition, potential unmeasured confounders such as smoking intensity, BMI, and comorbid conditions were not included in multivariable models due to data limitations and the risk of overfitting. The absence of longitudinal follow-up also prevented assessment of FAR dynamics and disease progression.

Future studies should validate these findings in larger, multicenter prospective cohorts and explore integration of FAR with radiological scoring systems or additional biomarkers to improve risk stratification and monitoring in occupational lung diseases.

## CONCLUSIONS

The authors' study demonstrates that FAR is positively and independently associated with pneumoconiosis, while albumin and blood lead did not retain significance after adjustment. Patients with pneumoconiosis exhibited higher FAR values alongside elevated fibrinogen and lower albumin levels, reflecting systemic inflammation related to occupational dust exposure. Given the modest odds ratio and moderate discriminative performance, FAR should be considered a supportive and complementary biomarker rather than a stand-alone diagnostic tool. Future multicenter prospective studies with larger cohorts are warranted to validate these findings and further clarify the role of FAR in occupational lung disease surveillance.
